# Understanding the Needs and Experiences With Health Services of Gay and Bisexual Men (GBM) Who Engaged in Chemsex During the First Year of the COVID-19 Pandemic in Quebec, Canada

**DOI:** 10.1177/10497323241302973

**Published:** 2024-12-15

**Authors:** Maxi Gaudette, David Ortiz-Paredes, Adam Bourne, Yannick Gaudette, Jorge Flores-Aranda, Rod Knight, Olivier Ferlatte

**Affiliations:** 1 Département de médecine sociale et préventive, École de Santé Publique, 248214 Université de Montréal, Montréal, QC, Canada; 2 Centre de Recherche en Santé Publique, Université de Montréal et CIUSSS du Centre-Sud-de-l’Île-de-Montréal, Montréal, QC, Canada; 3 Australian Research Centre in Sex, 2080 Health and Society (ARCSHS) La Trobe University, Melbourne, VIC, Australia; 4 234164 École de travail social de l’Université du Québec à Montréal (UQAM), Montreal, QC, Canada; 5 UQAM, Chaire TRADIS – Trajectoires, Diversité, Substances, Montreal, QC, Canada

**Keywords:** health crisis, party n’ play, sexualized drug use, MSM, online services, health needs

## Abstract

Gay and bisexual men (GBM) engaging in chemsex can face various health and well-being-related challenges, the extent of which remains unknown given the limited research in the context of the COVID-19 pandemic. This paper examines the pandemic impacts on the health needs of GBM who engaged in chemsex and their experiences with related services. We applied interpretive description to produce knowledge with direct implications for improving practices and policies. Data were collected between July 2020 and January 2021 using in-depth semi-structured interviews with eight key informants who held chemsex expertise and 13 GBM with chemsex lived experiences. Interviews were transcribed, anonymized, and analyzed thematically, yielding three interrelated themes. First, participants argued that the COVID-19 public health response was heteronormative and moralizing, reinforcing feelings of shame among GBM who engaged in chemsex and further isolating them. This added a layer of stigma and exposed them to increased drug-related risks by obstructing harm reduction practices. Second, participants contended how the pandemic worsened the scarcity and shortcomings of chemsex-specialized services. The mandatory shift to online services made it harder to form meaningful therapeutic relationships, especially given the unique sensitivity and stigma associated with chemsex, further heightened during the pandemic. Third, this online shift simultaneously facilitated access to personalized and culturally sensitive care, especially for those with less urgent needs. Our findings’ implications emphasize the importance of adopting a comprehensive approach in chemsex care, integrating both in-person and online methods, to counteract health iniquities reinforced by the pandemic and the institutional responses to it.

## Introduction

Chemsex, also known as party and play (PnP), refers to sexualized poly-substance use primarily by gay and bisexual men (GBM) and other men who have sex with men to facilitate, prolong, or enhance sexual encounters, among other reasons. Many scholars and community actors stressed the cultural aspects of chemsex by highlighting the unique set of norms, behaviors, and circumstances that distinguished chemsex from other sexualized substance use (Drysdale, 2021; Florêncio, 2021; Stuart, 2019). For example, chemsex is often practiced in groups at private parties or bathhouses using specific substances, including crystal meth (methamphetamines), mephedrone, GHB/GBL (gamma-hydroxybutyric/butyrolactone acid), and ketamine (Maxwell et al., 2019). Chemsex is also situated within a specific historical context where GBM have faced ongoing stigmatization of their sexuality and recreational substance use, further compounded by the HIV/AIDS epidemic (Race, 2015; Stuart, 2019). Chemsex has become a significant public health concern precisely due to its association with the transmission of HIV and other sexually transmitted and blood-borne infections (STBBIs) (Pakianathan et al., 2018; Rosińska et al., 2018; Rusow et al., 2017).

A systematic review found that chemsex offers GBM benefits beyond improved sexual performance and pleasure (Lafortune et al., 2021). Engaging in chemsex can serve as a coping strategy for stress, anxiety, loneliness, and stigma. It can also enhance social connections by fostering emotional closeness, acceptance, and belongingness. This echoes some studies tying chemsex with better mental health outcomes, including lower odds of depression (Field et al., 2023) and higher levels of resilience (J. Power et al., 2018). This literature challenges more common associations of chemsex to dependence, mental health and psychosocial issues, such as depression, psychosis, isolation, multilayered stigmatization, financial complications, etc. (Bourne et al., 2014; Li et al., 2021; Moreno-Gámez et al., 2022; Souleymanov et al., 2020; This et al., 2023). These contradictions highlight the diverse realities of GBM engaging in chemsex and, consequently, the diversity of their needs. These needs, at the crossroads of sexuality and substance use, include STBBI testing, psychosexual therapy, substance use treatment, community support, and harm reduction services (Bourne et al., 2015; Gaudette et al., 2022). Addressing these needs is challenging because many services are not designed to tackle the intertwined issues of sexuality and substance use concurrently. Furthermore, the predominantly biomedical approach to sexual health and substance use overlooks the social and cultural contexts influencing chemsex practices and well-being (Nagington & King, 2022). This is an issue in the province of Quebec, Canada, where the present study was conducted. Healthcare in Canada falls primarily under the jurisdiction of provincial and territorial governments (Marchildon et al., 2020). In Quebec, services accessed by GBM engaging in chemsex are fragmented, operating in silos and failing to address their complex needs (Dumas, 2013; Dumas et al., 2018; Flores-Aranda et al., 2019; McKirnan et al., 2013). This fragmentation is compounded by a lack of training on chemsex-related issues among mainstream healthcare professionals, leaving them with limited knowledge of both chemsex and GBM’s sexual health. Grassroots and community-led movements have been at the forefront of the development of chemsex-specialized services in Quebec, which remain limited. These services, which include harm reduction, mental and sexual healthcare, and peer support, are primarily offered by community-based organizations or sexual health clinics but always risk shutting down due to precarious funding (Cri de Ralliement Chemsex, 2024). Free or low-cost services tailored to chemsex are scarce and concentrated in the province’s largest urban center, Montreal, while private healthcare options are prohibitively expensive. The lack of formal integration with primary healthcare creates significant barriers for individuals navigating between stigmatizing general medical services and inaccessible or costly specialized care.

The coronavirus disease (COVID-19) pandemic exacerbated health and social inequities in Canada and elsewhere, disproportionately affecting vulnerable populations (CIHR, 2021; Karmann et al., 2024; Saalim et al., 2021), including GBM (Gaspar et al., 2021; Philpot et al., 2021; Souleymanov et al., 2023). Among GBM, those engaging in chemsex may have been even more impacted by the pandemic, given the psychological and social challenges linked to the practice, yet research exploring these consequences remains limited to date.

In Quebec, the COVID-19 health emergency was declared on March 13, 2020. As COVID-19 cases increased, the government implemented progressively stricter measures to curb virus transmission. These included enforcing social distancing, closing public places, banning indoor and outdoor gatherings, restricting travel, issuing stay-at-home orders, curfews and lockdowns, etc. On March 24, 2020, the Government of Quebec mandated the closure of all in-person services except essential until April 13, which was then extended until May 4, 2020 (INSPQ, 2022). In reaction to these measures, a shift toward virtual health services occurred. Research among GBM points to worsened health outcomes as a result of unaddressed sexual and mental health needs during the pandemic (Daroya et al., 2024; Souleymanov et al., 2023), but such findings regarding chemsex communities are not available.

Existing research on chemsex during the COVID-19 pandemic has mainly focused on its prevalence (see Connolly et al., 2023; Mugglin et al., 2023; Reyniers et al., 2021), reasons for engaging in chemsex during the pandemic and HIV-related risks (Chone et al., 2021; de Sousa et al., 2020; Hyndman et al., 2021). These studies reflect the tendency in the chemsex literature to focus on risk (Møller & Hakim, 2021). Particularly overlooked in this body of research are the interactions between GBM’s health needs and their access to health services resulting from the pandemic. This study aims to fill that gap by answering the following research question: *How did the first year of the COVID-19 pandemic impact the health needs of GBM who engaged in chemsex and their experiences with related services in Quebec, Canada?* Shedding light on the challenges GBM who engaged in chemsex faced during the pandemic can contribute to refining existing services and guiding decision-making in future pandemics.

## Methods

### Study Design

This study is grounded in the rigorous tradition of interpretive description (Thorne, 2016), a flexible approach that aims to produce evidence that has direct implications for improving practices. Thorne et al. (2004) suggest collecting data from different sources to give them more context and to avoid overemphasizing one-sided perspectives. We, therefore, purposefully collected data among two sources with different types of knowledge, that is, professional expertise (key informants—KIs) and lived experiences (GBM who engaged in chemsex), to have a broader understanding of the impacts of the pandemic.

### Recruitment and Participants

Our research team first met to identify a list of potential KIs with diverse professional backgrounds and extensive knowledge of chemsex. We looked for individuals who, because of their clinical, therapeutic, or social work, were in direct contact with GBM who engaged in chemsex before or during the pandemic. We invited eight KIs by email to take part in this study. Their demographic characteristics are summarized in Table 1. We prioritized recruiting KIs first to refine, based on their insights, our interview guide for the GBM who engaged in chemsex. Leveraging the KIs’ expertise and proximity to GBM who engaged in chemsex, we requested they help disseminate the recruitment posters among men who might be interested in participating. The recruitment material was also shared on social media (Instagram and Facebook), within community-based organizations, sexual health clinics, and the research team’s networks. The unprecedented circumstances of COVID-19 challenged the recruitment of participants. In this context, we prioritized accessibility and feasibility, relying primarily on online methods that complied with public health restrictions and the guidelines set by our university and ethics committee. We did not intentionally develop recruitment strategies to diversify our sample, a limitation we return to in the Discussion section. Potential participants meeting the inclusion criteria (i.e., being 18 years or older; being gay, bisexual, queer, or a man who has sex with men; and having chemsex lived experiences) were asked to contact the research team to schedule an online interview. A total of 13 GBM who engaged in chemsex in Greater Montreal were interviewed and received a 25$ compensation. All men had engaged in chemsex since the pandemic or shortly before. At the time of the interview, some were still engaging in chemsex while others had stopped or were trying to. Their socio-demographic characteristics are included in Table 2.

**Table 1.** Key Informants’ (*N* = 8) Socio-Demographic Characteristics.

Age	
30–39	5
40–49	1
60–69	2
Sexual orientation	
Gay	6
Queer	1
Heterosexual	1
Race/ethnicity	
White	8
Occupation	
Sexual health clinic	2
Mental health professional	3
Community worker/activists	2
Outreach worker	1

**Table 2.** GBM Who Engaged in Chemsex (*N* = 13) Socio-Demographic Characteristics.

Age	
20–29	2
30–39	4
40–49	4
50–59	3
Sexual orientation	
Gay	11
Bisexual	2
Race/ethnicity	
White	11
Latino	2
Substance use (past 2 months)	
Crystal meth	9
GHB/GBL	9
Cocaine	6
Ketamine	3
Speed (amphetamine)	3
None (except cannabis)	2

### Data Collection

Data were collected using semi-structured interviews of 60–90 minutes between July 2020 and January 2021. After receiving informed consent verbally, we conducted interviews online via Zoom in accordance with social distancing measures. Interviews were conducted in French (Quebec’s official language) by the first author and another graduate student trained in qualitative data collection. The interviews with KIs aimed at gathering information regarding the consequences of the pandemic for GBM who engaged in chemsex as well as specific challenges and difficulties they might have faced. The interviews with GBM who engaged in chemsex explored their experience with health services, including those offered by sexual health clinics, psychosocial therapists, sexologists, social workers, support groups, and community-based organizations. Topics discussed also included social distancing, confinement measures, substance use, and sexual, mental, and physical health during the pandemic. Interviews were transcribed verbatim to facilitate the analysis. The verbatim were then anonymized, and participants were given pseudonyms to protect their anonymity.

### Data Analysis

The data was analyzed with an inductive thematic approach using NVivo (Braun & Clarke, 2006). The first author and a research assistant first read participants’ transcripts thoroughly to get familiar with the data and then met virtually to discuss initial ideas regarding the codebook. Second, they worked collaboratively to generate initial codes and noted their meaning. The research team convened to discuss and combine codes into a comprehensive codebook, which was applied to all interviews. Third, codes related to health needs and services were extracted and analyzed by comparing their meaning to identify patterns (themes) answering our research question. Fourth, we returned to the participants’ transcripts to review whether the themes properly reflected the data. At this step, we reworked the themes by dividing or collapsing them into each other. Fifth, we further refined and defined each theme to clearly capture its essence. Sixth, we finalized the analysis throughout the writing of this article, selecting compelling extracts from participants’ transcripts, to portray the overall story the themes tell about the data.

## Results

For clarity in detailing the results, we divided health services into two categories. The first category refers to services that are more general GBM health services that are often used by both those who do and do not engage in chemsex, including STBBI testing, HIV prevention (PrEP and PEP) and treatment (ART), and harm reduction services. The second category refers to chemsex-specialized services offering support or treatment to people who have substance use or chemsex-related issues. Our in-depth analysis reflected three themes, which together help understand the impacts of the COVID-19 pandemic on the health needs of GBM who engaged in chemsex as well as their experience with related health services. These themes are supported and illustrated by quotes (translated from French) from both KIs and GBM who engaged in chemsex (referred to as those with “lived experience”).

### A Moralizing Response to COVID-19 Exposing GBM Who Engaged in Chemsex to Heightened Health Risks

Participants described the public health response to the COVID-19 pandemic as “very heteronormative” (Irène, KI) as it did not take into consideration the needs of GBM who engaged in chemsex. Instead, public health messaging was moralizing for these men and exposed them to increased health risks. By heteronormative, participants argued that the strategies put in place to prevent onward transmission of COVID-19 (such as stay-at-home orders and social distancing) were grounded in assumptions about interpersonal relationships and traditional family structures, conflicting with chemsex realities. Mathieu (KI) expressed his concerns regarding the heteronormative response to COVID-19 neglecting GBM who engaged in chemsex as follows:

My concern is that the public health discourse right now applies to ordinary people: “dad, mom, and two kids.” And if even the slightest bit you step outside of that framework, [you’re left behind]. It seems like there’s a lack of nuance. […We should] be careful with “one policy fixes everything,” it doesn’t work.

The implications and consequences of social distancing differed for GBM who engaged in chemsex compared to nuclear families who could maintain social bonds within the household and, for couples, meet their sexual needs. Many participants argued that public health measures negatively impacted GBM who engaged in chemsex by overlooking their need for sexual and social interactions. While these concerns may not have been exclusive to GBM who engaged in chemsex, they were intensified for this community given the complex interplay of sexuality, substance use, and sociality in shaping their well-being.

Many participants argued that expecting GBM who engaged in chemsex to refrain from practicing chemsex indefinitely is not realistic nor reasonable, especially considering how sexuality and substance use are inextricable for many of them. Mathieu (KI) explained that the public health measures and their incongruence with harm reduction principles were particularly unrealistic and damageable for those with substance dependence:

At first, even community organizations like [names anonymized], doctors and social workers, were like ... “stop everything, do nothing, and it’s a no. The answer is no!” That wasn’t a well-thought-out measure. It didn’t help anyone using drugs or dealing with these issues because, come on, it’s not realistic. It’s like saying to an alcoholic, “Stop drinking today! Just stop drinking.” It doesn’t work. That’s not how it works. So, that person ends up being even more isolated, turns around, and starts using in crooked [unsafe] ways. So, harm reduction sort of fell by the wayside.

Important in Mathieu’s (KI) and other participants’ narratives was how the unrealistic restrictions had isolating effects on GBM who engaged in chemsex, which exposed them to potential harm. For example, the confinement, curfew, and other social distancing measures exposed GBM who engaged in chemsex to increased substance-related risks, including overdose and death. One participant spoke about a friend who had died a few weeks into the pandemic of what he believed were substance-related issues aggravated by the pandemic. William (lived experience) shared a personal story to illustrate how social distancing measures worsened the risks associated with substance use. He explained a time when he was unable to support a chemsex friend in need, that is, by obstructing harm reduction practices that make substance use safer, such as using substances in the presence of others, mutual oversight for safety, monitoring substance intake, and assisting safe injection:

If it’s 9PM [when curfew starts] and my friend isn’t doing well, I can’t even go and see him. The other day, someone was here explaining to his friend over text [message] how to shoot up [inject himself] properly. Whoa! ... He was stressing because he was saying, “Damn, he wants to do it, and it’s his first time and I can’t go because of the lockdown.” And then they were talking by text, but what if something happens ... I don’t know, like if someone wipes out [loses consciousness]?

In addition to the risks detailed by William, participants argued that public health measures were moralizing and led GBM who engaged in chemsex to experience elevated levels of stigma, whether that be judgment, shame, or guilt, for engaging in chemsex during a health crisis. At the beginning of the pandemic, most community-based, health and governmental organizations had repressive approaches toward sexual encounters, which participants harshly felt. Irène (KI) explained that the widely spread message forbidding sexual encounters imposed a moral judgment on GBM who engaged in chemsex that was highly distressing:

The first weeks of COVID, it was shame! We were smearing shame. It was like, “If you don’t do this, you’re no good, and if you do that, you’re good.” We were sending out [such] messages … so those who fell between the two, well could be judged ... if you’re using drugs and sleeping with guys, well you’ve fallen into the wrong checkbox. And shame is toxic. That’s the problem.

These judgmental messages also circulated within the GBM community, especially on dating and hook-up apps. Participants reported receiving messages like “You’ll catch COVID, and you’ll give it to everybody!” (Karl, KI) or “Do you realize what you’re doing?” (Paul, lived experience), which they interpreted as reproachful or hateful and that resulted in feelings of shame for meeting sexual partners during the pandemic. Failing to adhere to public health measures added another layer of stigma to the already present judgments about chemsex practices. This further isolated individuals, and made accessing services more complicated, as explained by Mathieu (KI):

Sex on crystal meth comes with a certain level of stigma and shame, and now we’ve added another layer of stigma and shame. So, if you dare to have a sex life, or if you dare to have fun during a pandemic, you’re even more shameful. So, we notice that people hide more, but that doesn’t mean they consume less, it just means we see them less.

Participants suggested that instead of being told to avoid sexual encounters, they would have benefited from COVID-19 prevention strategies that were adapted to the realities of chemsex. When asking Jean (lived experience) about his needs during the pandemic, he emphasized the importance of harm reduction approaches. He suggested offering practical advice to reduce the risk of COVID-19 transmission without prohibiting sexual activities:

That’s it, [harm reduction approaches]. Exactly. For example, it could be telling people, like “Avoid kissing each other; give each other oral sex instead.” It might sound silly, but at the same time, maybe it helps. There are probably ways to approach sexuality that are safer than others [during a pandemic], but without resorting to prohibition.

Many participants, like Jean, were preoccupied with preventing the transmission of COVID-19 but struggled to find ways to do so while also addressing their social and sexual needs. These men could have benefited from sex-positive and culturally sensitive information on how to engage in chemsex in the safest way possible.

Participants also criticized the closure of all services deemed non-essential. They argued this closure reflected and reinforced heteronormative assumptions of what is considered essential, neglecting the sexual health needs of GBM who engaged in chemsex. Karl (KI) lamented about the closure of sexual health clinics as they are often the only entry point for these men into the healthcare system. He explained that the closure of sexual health clinics had particularly negative consequences for sexual health as STBBI testing was no longer available, which exposed them to related harms: “I wouldn’t have closed the clinics completely like they did, because by closing essential services to these guys … it’s like saying, ‘You don’t exist anymore; we won’t take care of you anymore.’” Men who engaged in chemsex who relied on these services pre-pandemic suddenly saw their needs ignored during times that were already isolating, uncertain, and difficult.

In sum, the “one-size-fits-all” response to the pandemic prioritized the prevention of the virus at the expense of sexual and mental health needs that fall outside of a heteronormative understanding of well-being. This moralizing response weighed heavier for GBM who engaged in chemsex, further stigmatizing them and exposing them to related risks.

### COVID-19 Exacerbating the Scarcity and Shortcomings of Chemsex-Specialized Services

In addition to the closure of general GBM health services, there was a common feeling among participants that the pandemic made evident the limitations of the chemsex-specialized services, specifically individual therapy and group support. Many participants argued that the pandemic simultaneously added stress on these services while complicating their access to GBM who engaged in chemsex, marked by a shift from in-person to online care provision. According to participants like Jean (lived experience), finding appropriate services for chemsex-related issues was already challenging before the pandemic, and this was further complicated due to COVID-19 public health measures: “Regardless of the crisis, resources are insufficient or inadequate … but COVID may have worsened something that wasn’t strong before.”

Participants denounced the already limited number of health professionals with chemsex expertise in Quebec and how the pandemic added stress to these resources as demand for support was high among GBQMC during this period. Christian, a KI offering chemsex-specialized therapy, shared how the pandemic introduced new challenges to those serving this community, such as changes to maintaining clients’ anonymity and confidentiality while working from home. These challenges required significant adjustments in how services were delivered, consuming more time and resources, and ultimately led health professionals like Christian to turn away new clients:

COVID-19 has already disturbed a lot [of things]. What I did for the summer is that I stopped taking on new clients, which is a bit heartbreaking because there is a lot of demand, but there is also the quality of service.

Participants expressed concerns regarding the mandatory shift from in-person to online service in terms of contributing to the inadequacy of chemsex-specialized services. They highlighted how online services are not accessible to all GBM who engaged in chemsex as it necessitates a device, internet connection, and sufficient understanding of the technology (such as teleconferencing software). This could be particularly challenging for those in precarious situations like unstable housing. Hugo (KI) was concerned that the transition to online services would exacerbate these barriers: “They [GBM who engaged in chemsex] also need access to a phone … So that’s why I’m telling you, what about the more vulnerable people?” Similarly, some participants viewed online services as less effective and less accessible for those who had not previously used them in person. Participants were highly attuned that an in-person connection fostered strong rapport, which was paramount to effective care and productive therapeutic relationships. Reflecting on his first experience attending a 12-step support group, which rapidly moved online due to the pandemic restrictions on gatherings, Luc described how meeting other individuals in person was key to him feeling welcome, seen, and heard:

When I walked into the room and met people for the first time, that’s what helped me the most at the beginning. I don’t know if you can feel that support as strongly when you have a Zoom meeting online compared to when you go in person with people who are there: you feel the warmth and energy emanating from them, and you physically see how much better they are doing or whatever. I think online, it’s easier to pretend that you’re not doing well. I don’t know to what extent new people who arrive online see that it’s possible to recover.

Participants shared the importance of face-to-face interactions in creating genuine connections, especially when initially building authentic rapport. They felt that online services made it harder to form meaningful therapeutic relationships, describing these virtual interactions as less personal, authentic, and engaging. It is important to situate this issue in the context of chemsex as an extremely sensitive and stigmatized practice, even more so during the pandemic. Talking to an unfamiliar person via an unfamiliar technology can challenge these men’s ability to develop rapport and to talk openly and honestly about their experiences and needs. This was reflected in William’s (lived experience) mistrust of online services, doubting their effectiveness:

It’s not the same behind a camera. You don’t have the person in front of you, you don’t have the intimacy, you don’t have the feeling of confiding. Right now, I’m in my room … I’m in the same place where I use drugs to talk about my drug use. I don’t feel like it.

For William and others, online services were inadequate because they were necessarily accessed from home, which is also where they engaged in chemsex. These participants were concerned that receiving services in the same environment where they use substances was ineffective as it might not offer enough separation to reflect deeply on their experiences with chemsex. This shortcoming was similarly reflected in a discussion with Jerry (lived experience) when he criticized online services for making it harder to commit to the therapeutic alliance with his therapist:

Once, I missed his call [from my therapist]. It doesn’t hold me … accountable … if I had that appointment [in person], I would have taken the time to prepare myself, to make sure I was there on time, [plan] my subway ride and all that ... and now it’s just like “Oh well, she’ll call me, and I can use drugs, she won’t know a thing.”

According to Jerry, receiving services at home prevented him from fully committing to the therapeutic process. It was also possible for him to use substances during his sessions and take them less seriously despite being aware of how this could negatively impact the service effectiveness. Participants also noted that remaining engaged and focused during online sessions was harder, making them less adequate compared to in-person services. Christian (KI) reflected on how these added challenges negatively impacted his relationship with clients:

What’s tricky with guys who use crystal [meth] is their focus, attention, concentration, memory, commitment. All of these things are really challenging, especially when substance use is prevalent, and there’s little sleep, and there’s disorganization in [their minds] there that creates a lot of unmanageable emotions … So, attending sessions through a screen makes it even harder to focus.

Evident in Christian’s and other KIs’ narrative is the importance of being present to captivate, motivate, or engage clients who use crystal meth, which highlights online services’ shortcomings for some GBM engaging in chemsex. These challenges were more important for men with greater mental health and substance use issues, leading, for example, to sleep deprivation and lack of motivation during therapy. Many KIs worried about the impacts of the shift to online service for men at the intersection of these vulnerabilities, specifically regarding crisis interventions. Christian (KI) spoke to this, worrying that a crisis intervention with a man engaging in chemsex in a particularly vulnerable state can cause them more harm:

It’s very unsettling to see someone in such a bad shape, in withdrawals, on the verge of toxic psychosis, on the other side of the screen. What type of intervention do you do? What will put him at risk? What happens on the other side [of the screen] when we turn off the computer? So, there’s my desire to help them, but also my concern that the services we can offer right now, at least what I can offer, are not optimal because … well, this person has been isolated for months, they’ve been slamming [injecting crystal meth] for months, they don’t need online therapy for exploring their sexuality. They need something much more grounded and to be received with compassion, something more present. (Christian, KI)

KIs articulated that the online approach frequently proved insufficient in the context of the pandemic, particularly for men facing greater adversity in terms of social isolation, mental health, and substance use. According to them, being physically present was fundamental to expressing genuine emotions of care and compassion toward their clients and adapting the intervention to their clients’ physical and emotional state, even more critical during a crisis. Without these capacities, some KIs limited the type of intervention they offered, ultimately contributing to the scarcity of chemsex-specialized services.

In sum, the pandemic reinforced the scarcity and inadequacy of chemsex-specialized services as the unprecedented context generated new issues and barriers both for healthcare providers and GBM who engaged in chemsex. The mandatory shift to online services was in many ways incompatible with some participants’ need for in-person interactions and face-to-face connections deemed essential to the quality of care. Some of these services, like crisis intervention, could consequently not be offered online, which added pressure on the overwhelmed resources, ultimately further limiting service provision.

### Online Services Offering New Possibilities in Terms of Safety and Tailored Care

Despite participants’ critical stance on the adequacy of online services for GBM who engaged in chemsex, they also provided a nuanced perspective highlighting the advantages of this digital transition. Participants particularly emphasized enhanced safety and the opportunity for more personalized care. While new, the possibilities of online approaches were met with optimism by many participants who saw the potential to bridge some of the previously mentioned gaps in chemsex care. KIs discussed the potential of online approaches in responding to the lack of combined knowledge on substance use and sexuality by more easily connecting health professionals with this respective expertise. For Hugo (KI), the pandemic context that mandated working virtually could foster and legitimize collaborations in real time between health professionals that might not have occurred otherwise: “So this kind of technological turning point … I think, is going to have great repercussions and maybe, we’ll hope … be able to set up remote teams.” Remote teams were particularly promising for Hugo, who expressed frustration over services working in silos pre-pandemic—an important barrier to addressing the needs at the intersection of “chem” and “sex.” He continued:

Let’s say [a GBM who engaged in chemsex] goes to see his addiction therapist, and the addiction therapist is like “Okay, my competencies end here, I would need the support of a sexologist or another specialist,” well [the therapist] can call them and continue in teleconsultation.

Though Hugo had not yet witnessed such collaborations, his account exemplifies the possibility enabled by the rise and legitimization of online services in addressing the complex needs of GBM who engaged in chemsex. It showcases how professionals from different domains can come together more easily to provide specialized care as geographical barriers or logistical challenges may be overcome through virtual communication. In this context, health professionals can tap into an interdisciplinary network of specialists to offer more tailored and comprehensive assistance to GBM engaging in chemsex.

Some participants with chemsex lived experiences also shared positive feedback after their experience utilizing online services, particularly highlighting the overall simplicity, ease of use, and ability to obtain health services from the comfort of home. Such was the case for Sergio (lived experience), who started seeing his therapist virtually since the pandemic began. When asked about his experience regarding this change, he explained, “I find it easier, not having to travel and all that. I think this way it’s simpler, and we still achieve the same goal.” Similarly, Adrian (lived experience) provided an example of how having to get to therapy before the pandemic amidst traffic would induce stress and negatively impact his sessions. Doing therapy online meant he could avoid these feelings and gain more focus and, as such, derive more benefits from the therapeutical relationship:

[My sexologist appointments] were always in the evening. As soon as I finished work, I had to rush there, and there was always traffic, so I always arrived very stressed out, on edge, and losing it … Whereas now, it’s just like, I get up from my chair, I go to my couch, I turn on the computer; that’s it. So, I no longer have time to stress about traffic, and … now it’s going well. It’s still like that, and it’s going to stay like that, in my opinion.

Adrian’s comments underscore the significant impacts of one’s emotional and physical state in shaping the effectiveness of the therapeutic process. Some viewed accessing therapy from home as advantageous, offering a familiar, safe, and tranquil setting that facilitated healing. These benefits were also discussed in the context of support group meetings, such as 12-step programs. The shift to online meetings expanded the opportunities for men to access a broader selection of meetings, not limited to geographical locations or specific meeting times. A notable advantage of online services highlighted by some participants was the ability to remain anonymous, for instance, by choosing not to activate their camera during teleconference meetings. This feature was considered crucial within the context of chemsex, where stigma can be a significant barrier to seeking help. For example, Félix (lived experience) specified he was more comfortable assisting 12-step group meetings online: “For me, Zoom was good. I’m a pretty reserved guy, so being at home, with each person in their own space, suited me better than meeting everyone in a room. So, it worked out well.” Online spaces felt safer and more accommodating to some GBM who engaged in chemsex, like Sergio, who even desired to “continue like this,” that is, utilizing them after the pandemic subsided.

In sum, participants reflected on the opportunities arising from the pandemic in terms of service provision. The adaptability and safety afforded by online services facilitated a more comfortable, stress-free, and effective engagement for some participants, thereby enhancing the therapeutic process. This shift underscores the potential long-term benefits of integrating online service options within the broader framework of health and support services, suggesting a continued role for virtual platforms in offering flexible, accessible, and user-centered care beyond the pandemic context.

## Discussion

There is currently very limited literature on the consequences of the COVID-19 pandemic for GBM who engaged in chemsex as it relates to their health needs and their experiences with health services. Our study advances this emerging literature by capturing and comparing the perspectives of both GBM who engaged in chemsex and KIs working closely with them. Gathering viewpoints from service providers and users allowed for a comprehensive and triangulated understanding of how the pandemic impacted these men’s needs and experiences with health services. Our research identifies three interrelated themes that highlight the intricate challenges they faced in accessing health services amidst a moralizing public health response to COVID-19 that neglected their needs. In this context, the rise of online services created both challenges regarding accessibility and adequacy, and opportunities in terms of tailored care and safety. As we exit the COVID-19 pandemic, further understanding these issues, challenges, and opportunities can inform policymaking regarding future health crises and inform current service delivery related to chemsex, specifically in terms of online versus in-person models, to promote the best outcomes possible.

For many GBM, chemsex represents more than mere sexual activity. It encompasses feelings of belongingness, strengthens social connections, and contributes to mental well-being (Field et al., 2023; Lafortune et al., 2021). This supports participants’ perception of the moralistic and heteronormative response to the pandemic as rendering invisible the physical, mental, social, and sexual health needs of GBM who engaged in chemsex. Previous research has recognized the heteronormativity embedded within the COVID-19 response, which reinforced heterosexual models of kinship and negatively impacted sexual and gender minorities’ sense of belonging (Daroya, Gaspar, et al., 2023; Lucas et al., 2022). Our study contributes to these findings by drawing attention to the specific ways this response neglected the needs of GBM who engaged in chemsex, for example, the closure of services essential to these men, like sexual health clinics, while simultaneously exposing them to increased stigma and substance-related harm. Participants’ experiences showcase how this moralistic messaging was not only vehiculated by governmental officials but also endorsed, at the beginning of the pandemic, by GBM, community-based organizations, and sexual health clinics. Such messaging dampened or erased sex positivity and harm reduction, which are driving values of many community-based organizations and sexual health clinics catering to GBM. This underscores the pervasive influence of dominant discourse in marginalizing the needs of individuals when they diverge from this prevailing narrative. Our study suggests that GBM engaging in chemsex during the first year of the pandemic faced triple-stigma, that is, not only for their sexual orientation or substance use but also for not being able to follow public health measures. This is concerning considering the substantial body of literature on the multiple and interconnected negative health outcomes of stigma for GBM (Brener et al., 2023; Flentje et al., 2020; Pollard et al., 2018).

This study contributes to the existing body of literature on the paucity and inadequacy of services tailored to individuals engaging in chemsex (Bourne et al., 2015; Flores-Aranda et al., 2019; Nagington & King, 2022) by elucidating how the COVID-19 pandemic intensified these deficiencies. Participants made evident that the pre-pandemic challenges of accessing appropriate chemsex-specialized services were compounded by the restrictions put in place to prevent onward transmission of COVID-19. Some participants perceived online services as inadequate because of the lack of human connection and empathy, as observed in similar studies with GBM (Daroya et al., 2024) and with psychologists (Downing et al., 2021). Unique, however, to GBM who engaged in chemsex is the triple-stigma related to the practice, which might have negatively impacted their trust and comfort toward online services, thereby challenging the establishment of a therapeutic relationship. Participants also articulated that doing therapy at home, in the same environment where chemsex occurs, did not allow for enough distance to engage in therapy properly. This is not surprising as Downing et al. (2021) illustrate how the traditionally safe environment of in-person therapy can be compromised by interruptions and privacy concerns from the client’s home during online sessions—substance-use-related stressors are exemplified within our study.

Amidst the challenges and limitations of online services, our findings also stress the new possibilities they offer in terms of safety and tailored care. Namely, KIs were interested in the potential of the shift from in-person to online service to facilitate collaboration between professionals with different expertise. These types of innovations and collaboration resulting from the pandemic have been shown in recent Canadian studies conducted with service providers working with sexual and gender minorities (McKenzie et al., 2023) as well as with people who use substances (Jauffret-Roustide & Bertrand, 2022).

We want to draw attention to the conflicting findings of this study concerning online services, as they might indicate deeper health inequities. First, online services were more accessible and efficient for men using these services pre-pandemic while simultaneously increasing barriers for those new to them or those without the technological means. Daroya et al. (2024) similarly noted the importance of pre-existing relationships with health professionals in facilitating the transition to virtual care. However, those with restricted technological access or literacy, which are often already at the intersection of other vulnerabilities, face additional barriers to responding to their health needs, as supported by a growing body of literature (Beaunoyer et al., 2020; Lai & Widmar, 2021). Second, receiving services from home had negative impacts on the therapeutic process, mostly for men with more serious substance use or mental health issues, while being beneficial, stress-free, and convenient for men with less immediate needs and who had previously a trust relationship with the service. These positive impacts on therapy were distinctly evident, underscoring the interconnected role of physical settings and therapeutic relationships in shaping participants’ experiences. Together, these tensions highlight that the transition to online services during the pandemic might have worsened health disparities within this community, contributing to the body of knowledge about health inequities arising from COVID-19 (CIHR, 2021; Karmann et al., 2024; Saalim et al., 2021). These findings have direct implications for practice and policy by highlighting that services need to consider the diversity within GBM engaging in chemsex in terms of degrees of vulnerability, especially in times of a health crisis. Our study suggests online services were less beneficial for those with a higher degree of vulnerability. To counteract these iniquities, services available to GBM who engaged in chemsex must encompass a variety of approaches, that is, incorporating both in-person and online methods to address the diverse health needs of individuals who have been rendered vulnerable not only by the pandemic itself but also by governmental responses to it.

Our findings have other implications for practice and policy. This study indicates that heteronormative messaging forbidding sexual encounters may not have effectively deterred GBM from engaging in chemsex during the pandemic. Instead, such messaging may have alienated them, leading to heightened stigma and isolation. This, in turn, could have contributed to increased substance-related harms, particularly overdose. Participants in this study voiced a need for information or strategies to mitigate risks that align with their realities inspired by harm reduction approaches and principles (see Pauly, 2008). Rather than outright forbidding men from engaging in sex, given that some will continue to do so, and that repressive discourse can exacerbate harm, it is crucial to find strategies that mitigate risks while also considering their social and sexual needs. Some sexual minority men in Canada have shown agency in creatively adapting their sexual behaviors to prevent COVID-19 transmission, including wearing a mask during sex, prioritizing regular partners (“sex bubbles”), having sex outdoors, avoiding physical contact by voyeuristic masturbation, asking for COVID-19 symptoms, status, and individual safety measures, and only having sex with individuals vaccinated for COVID-19 (Daroya, Grey, et al., 2023). These strategies are reflected in an awareness document by the New York City Department of Health and Mental Hygiene (2021), which also encouraged virtual sex, washing up before and after sex, and being “creative with sexual positions and physical barriers that allow sexual contact while preventing close face-to-face contact” (p. 2). Such governmental initiatives are a prime example of how health officials can promote inclusive and sex-positive messaging even in the context of the pandemic. Historically, civil society has played a crucial role in responding to public health crises, particularly among marginalized communities. A collaborative approach, which combines the strengths of civil society with those of formal healthcare systems, is essential to address the health inequities that the pandemic has exacerbated and that will likely persist beyond it. Building on the historical and ongoing successes of community-led responses to epidemics like HIV (see Logie & Turan, 2020), there is a clear opportunity for synergy between civil society and government efforts. Such collaborations can lead to the development of flexible, accessible, responsive, and inclusive interventions.

### Study Limitations

The following limitations should be considered in light of the study’s contributions. Our study suggests the need for technological means and literacy is a fundamental barrier to online services. This challenge is also inherent to our data collection method, given it occurred online. We were unable to capture the experience of those without internet or a device needed to take part in the study, limiting our results by excluding those in more vulnerable situations. Importantly, our study sample lacks racial diversity, as all but two participants were white, limiting our findings’ transferability. Both the Public Health Agency of Canada (2021) and scholars (Grey et al., 2023; T. Power et al., 2020) have argued structural racism and colonialism exacerbated the impact of the COVID-19 pandemic on Indigenous, Asian, Black, and other racialized communities, leading to worse health outcomes. The fact that these communities were hardly represented in our study circles back to how more marginalized groups were even harder to reach during the pandemic. Future research should take this into consideration and adopt recruitment strategies specifically targeting these groups, for example, by collaborating with community-based organizations that work closely with Indigenous, Black, or Asian communities. Finally, data was collected during the first year of the pandemic and, as such, is not representative of the entire COVID-19 period as restrictions, knowledge of the virus, and attitudes evolved throughout the pandemic.

## Conclusion

The COVID-19 pandemic has had far-reaching impacts, particularly on vulnerable populations, the full extent of which we are still coming to understand. For GBM who engaged in chemsex and the providers serving them, it is clear that their health needs were not met during the first year of the pandemic, and a large sentiment of abandonment was felt for this population. The unprecedented crisis created high stress levels for this population at a time when compassion and understanding were needed. Unfortunately, the pandemic exacerbated existing issues regarding the scarcity and adequacy of these services and might also have worsened health inequities within this community. This might be the result of a one-sided approach that mainly focused on preventing the virus from a heteronormative standpoint. As we know more about the virus and its disproportionate consequences for vulnerable groups, public health officials should be better prepared for future health crises to avoid further neglecting the needs of these groups. We call on policymakers to consider the role of sexual and mental health in future health crises since they are closely linked to physical health.
